# Analysis of Triplet Exciton Loss Pathways in PTB7:PC_71_BM Bulk Heterojunction Solar Cells

**DOI:** 10.1038/srep29158

**Published:** 2016-07-06

**Authors:** Hannes Kraus, Michael C. Heiber, Stefan Väth, Julia Kern, Carsten Deibel, Andreas Sperlich, Vladimir Dyakonov

**Affiliations:** 1 Experimental Physics VI, Julius Maximilian University of Würzburg, D-97074 Würzburg, Germany; 2 Institut für Physik, Technische Universität Chemnitz, D-09126 Chemnitz, Germany; 3 Bavarian Centre for Applied Energy Research (ZAE Bayern), D-97074 Würzburg, Germany

## Abstract

A strategy for increasing the conversion efficiency of organic photovoltaics has been to increase the V_OC_ by tuning the energy levels of donor and acceptor components. However, this opens up a new loss pathway from an interfacial charge transfer state to a triplet exciton (TE) state called electron back transfer (EBT), which is detrimental to device performance. To test this hypothesis, we study triplet formation in the high performing PTB7:PC_71_BM blend system and determine the impact of the morphology-optimizing additive 1,8-diiodoctane (DIO). Using photoluminescence and spin-sensitive optically detected magnetic resonance (ODMR) measurements at low temperature, we find that TEs form on PC_71_BM via intersystem crossing from singlet excitons and on PTB7 via EBT mechanism. For DIO blends with smaller fullerene domains, an increased density of PTB7 TEs is observed. The EBT process is found to be significant only at very low temperature. At 300 K, no triplets are detected via ODMR, and electrically detected magnetic resonance on optimized solar cells indicates that TEs are only present on the fullerenes. We conclude that in PTB7:PC_71_BM devices, TE formation via EBT is impacted by fullerene domain size at low temperature, but at room temperature, EBT does not represent a dominant loss pathway.

Over the last decade, significant developments in the field of organic photovoltaics (OPVs) have pushed power conversion efficiencies above 11% in the lab and up to 9% in modules^1^. Despite increasing competition from other emerging photovoltaic technologies, OPVs remain highly attractive due to their low carbon footprint, low energy payback time, and rapid, cheap manufacturing and deployment potential^2^. Improvements over the last decade have been largely driven by efforts to increase the open-circuit voltage by materials energy level tuning^3^,^4^. Increasing the effective bandgap of the donor-acceptor blend has been achieved mostly by developing semiconducting polymers with lower HOMO and LUMO energy levels that match better with fullerene-based acceptors.

Blends with a low LUMO-LUMO and/or low HOMO-HOMO energy difference (low energy offset blends) result in heterojunctions with weaker donating and accepting strength, and a primary concern has been whether or not exciton dissociation would still be efficient. Studies have shown that charge transfer between the donor and acceptor can still occur with a relatively small energetic driving force^5^,^6^,^7^,^8^,^9^. In addition, however, it was proposed that low energy offset blends should have a new loss pathway resulting from charge recombination to the now energetically favorable triplet exciton states in the donor or acceptor^10^,^11^,^12^,^13^,^14^. In some blends, triplet exciton formation has appeared to be a major and even dominant loss mechanism^5^,^9^,^11^,^14^,^15^,^13^,^14^,^15^,^16^,^17^,^18^,^19^,^20^,^21^,^22^,^23^, but in some others, it has been shown that even when energetically favorable, it can be largely avoided^7^,^24^,^25^,^26^,^27^. For OPVs to become a viable technology, the triplet exciton loss pathway must be minimized. By understanding the factors that dictate whether or not triplets form, we can update design rules for next generation materials and devices. This understanding may also provide valuable information for other organic optoelectronic devices where triplet excitons play a key role, including light emitting diodes and photodetectors, and for future organic spintronics applications.

In neat organic semiconductors, optical excitation creates predominantly singlet excitons, which have a spin multiplicity of zero. However, there are also lower energy triplet exciton states, which have a spin multiplicity of 1. Triplet excitons can form via several pathways: singlet intersystem crossing, singlet fission, and charge recombination. Intersystem crossing is usually slow (ns timescale)^28^,^29^,^30^ due to weak spin-orbit coupling^31^, and singlet fission is only significant under very high excitation density^32^,^33^. However, triplet exciton formation from charge recombination can be quite efficient. When two spin-uncorrelated nongeminate charge carriers recombine, theoretically 75% of recombination events should produce triplet exciton states. However, there has been controversy whether or not triplet exciton formation from injected charge carriers in neat materials follows simple spin statistics^34^,^35^,^36^.

In blends, questions still remain regarding which factors control whether or not triplet formation occurs and if so, whether or not it represents a major loss mechanism in high performance OPVs. In high energy offset polymer:fullerene blends where the intermolecular charge transfer (CT) states have a lower energy than the triplet exciton states, the triplet excitons that may normally form in the neat polymer by intersystem crossing are quenched due to the presence of the fullerene acceptors^37^,^38^,^39^,^40^. The analogous quenching behavior is also observed with excitons formed in the fullerene phase. However, in low energy offset polymer-polymer blends, triplet exciton formation can actually be enhanced relative to the neat materials^15^, and triplet exciton formation is thought to be mediated by spin mixing via charge separated states^11^,^14^,^17^. Nevertheless, in some polymer:fullerene blends, triplet formation does not appear to be a major loss channel, and the leading hypothesis is that delocalization of the CT states allows charge separation to kinetically outcompete triplet exciton formation^26^,^41^. Restricting the discussion to blends where triplet formation is energetically favorable, this model explains why polymer-polymer blends, which exhibit bound CT states, show major losses to triplet excitons^11^,^15^,^16^,^17^,^19^, while some high performing polymer:fullerene blends do not. Fullerene aggregation and order have been shown to be an important factor that promotes efficient charge separation in polymer:fullerene blends due to charge delocalization and enhanced hole mobility^18^,^42^,^43^,^44^,^45^. In several blends, when increasing the fullerene loading content^10^,^18^ and creating larger domains that presumably promote more delocalization^24^,^26^, triplet exciton formation is suppressed. However, recently in PCPDTBT:PC_71_BM blends, when the processing additive 1,8-octanedithiol (ODT) is added to coarsen the fullerene domains, the opposite effect is observed, and the triplet exciton yield increases^46^. As a result, the morphological factors that dictate whether or not triplet excitons are a dominant loss channel are still unclear.

In this study, we report on the effect of fullerene domain size on triplet exciton formation in PTB7:PC_71_BM blends at two different temperatures using photoluminescene (PL) and optically and electrically detected magnetic resonance (ODMR, EDMR) measurements. OPVs made with blends of poly[[4,8-bis[(2-ethylhexyl)oxy]benzo[1,2-b:4,5-b’]dithiophene-2,6-diyl][3-fluoro-2-[(2-ethyl-hexyl)carbonyl]thieno[3,4-b]thiophenediyl]] (PTB7) and [6,6]-phenyl-C71-butyric acid methyl ester (PC_71_BM) with the processing additive 1,8-diiodoctane (DIO) have achieved 7–9% power conversion efficiency^47^,^48^ with efficient charge separation and a reduced nongeminate recombination rate^49^. In this blend, the addition of DIO has been well-characterized to have a major impact on the fullerene domain size^47^,^50^,^51^. As a result, PTB7:PC_71_BM is an example of a high performing polymer:fullerene blend that will provide insight into the effect of processing additives on triplet exciton formation.

A number of previous studies have used transient photoinduced absorption spectroscopy (TAS) measurements to study triplet exciton formation. However, with TAS it can be difficult to decisively assign absorption peaks to each specific photo-excited species due to often broad overlapping absorption peaks coming from both the excitons and polarons present in the sample. In addition, excitation intensities significantly higher than standard 1 sun illumination are often needed to obtain a sufficient signal-to-noise ratio. Transient effects such as energetic relaxation can also cause transient behavior to be measurably different from steady state behavior. To obtain an unambiguous identification of triplet excitons in organic solar cells under standard operating conditions at steady state, a spin-sensitive technique such as ODMR or EDMR is preferable^21^,^52^,^53^,^54^,^40^. Taking advantage of the paramagnetic properties of the triplet exciton state, a magnetic field can be used to manipulate the system, and these manipulations can then be detected in order to determine the presence of triplet excitons in the film. At steady state, all excited states in the material are coupled together by a complex series of reaction pathways, including triplet formation:


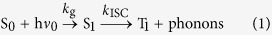



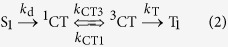


radiative recombination:


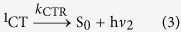



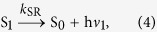


nonradiative exciton-exciton annihilation:


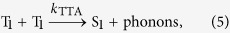



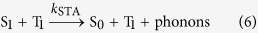


nonradiative exciton-polaron annihilation:


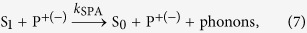



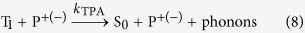


and nonradiative recombination:


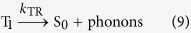



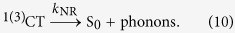


By applying a resonant microwave field and the appropriate magnetic field to the sample, one can induce Zeeman sublevel transitions within the triplet exciton manifold that then modify the overall triplet-triplet annihilation rate (*k*_TTA_)^55^,^56^,^57^, triplet-polaron annihilation rate (*k*_TPA_), triplet relaxation rate (*k*_TR_), or intersystem crossing rate (*k*_ISC_). Increasing any of these rates, drives the system of reactions forward, resulting in an increased steady state photoluminescence yield and a reduced charge carrier density. As a result, an increase in optical emission or a decrease in the solar cell open-circuit voltage *V*_OC_ induced by the applied magnetic field and resonant microwaves can be used to determine whether or not triplet excitons are present, and a comparison between samples can be used to determine the relative triplet yield.

## Results

**Figure 1:**
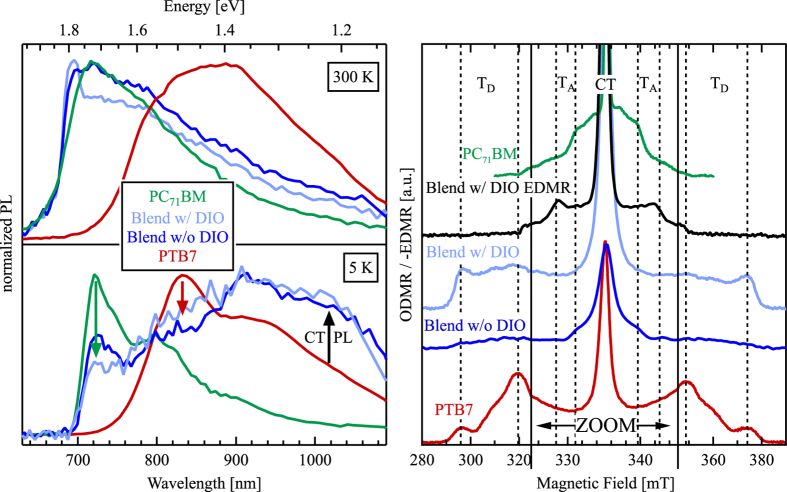
Left: Normalized PL spectra of neat PTB7 (red), neat PC_71_BM (green), PTB7:PC_71_BM blend without (blue) and with DIO (light blue) at *T* = 300 K (top) and *T* = 5 K (bottom). In the lower graph the spectral regions of interest have been marked with arrows to highlight emission from PC_71_BM (green) and PTB7 (red) singlet excitons, as well as singlet CT states (black). Right: ODMR spectra of the same materials at *T* = 5 K together with an EDMR spectrum of a solar cell with DIO at *T* = 300 K. The magnetic field range around the central CT state peak (325–345 mT) is zoomed in, and the peaks and shoulders of the PTB7 triplet excitons (T_D_) and the PC_71_BM triplet excitons (T_A_) are marked with dashed lines.

To identify which formation mechanism dominates in blends with and without DIO, PL quenching measurements were first performed at ambient and cryogenic temperature and are shown in Fig. 1 (left). In the neat PTB7 film (red), strong PL in the near infrared was observed with a broad peak at around 830 nm (1.5 eV), and the emission from neat PC_71_BM (green) is at higher energies, peaking at 710 nm (1.75 eV). The change in temperature mainly influences the vibronic substructure of the emission spectra by broadening the detailed features into one broad peak.

In both blend films, the overall PL is strongly quenched to ≈13% percent at 300 K and to less than 2% at 5 K relative to the neat material films. At 300 K, the PL spectra of both blends closely resemble that of the neat PC_71_BM film, with only a slight contribution from PTB7 emission at higher wavelengths. However, at 5 K the situation is more complex: Emisison from PC_71_BM is still clearly visible (marked by the green arrow), but no clear PTB7 emission can be discerned (red arrow). Instead, a broad featureless emission band at wavelengths >900 nm emerges (black arrow), which we tentatively assign to singlet CT state PL. Especially at 5 K, the addition of DIO has a clear impact on the residual emission from the fullerene phase. The blend without DIO has been well-characterized as having large, mostly pure fullerene domains^47^,^50^,^51^, consisting of smaller (ca. 20–60 nm wide) fullerene clusters^58^. The PL quenching results (blue) are consistent with this morphology due to the significant residual emission from the fullerenes at 1.7 eV. Processing with DIO primarily redistributes the fullerene clusters in the blend and thus increases the interfacial area with the PTB7-rich phase. In accordance with this picture, the fullerene emission from the film with DIO (light blue) is less than that of the film without DIO (dark blue). As a result, the blend samples with and without DIO exhibit PL quenching behavior that confirms the expected morphological differences characterized in previous studies.

Triplet exciton formation was then determined by the ODMR (5 K) and EDMR (300 K) measurements shown in Fig. 1 (right). The spectra for neat PTB7 (red) and neat PC_71_BM (green) demonstrate the fingerprint for triplet excitons in each material. For the neat PTB7 film, the distinguishing features are two pairs of peaks labeled T_D_ that correspond to Δ*m*_*s*_ = ±1 sublevel transitions: an outer pair at 296 and 374 mT and an inner pair at 320 and 349 mT. In the PC_71_BM film, the characteristic feature is a much narrower pair of peaks and shoulders at 331 and 340 mT and an outer pair at 328 and 343 mT labeled T_A_. The observed features match literature values for the triplet excitons’ zero-field splitting anisotropy^59^,^14^. In all spectra, there is also a central peak at 335 mT previously assigned to CT states^60^. Several of the outlined reaction pathways including triplet-triplet or triplet-polaron annihilation and ^1−3^CT ISC contribute to this peak’s intensity^60^,^61^. Therefore, this feature cannot be used to clearly identify the formation of triplet excitons and will mostly be ignored in this discussion.

Moving now to the blends, the film without DIO (blue) shows very low intensity PTB7 triplet exciton peaks and a weak signal coming from PC_71_BM triplet excitons. In contrast, the blend with DIO (light blue) does not exhibit the PC_71_BM triplet exciton signal, and an 8–10 times stronger signal coming from PTB7 triplet excitons emerges. The narrow peak from CT triplet states also increases by ≈4 times. We also note that ODMR signals of T_D_ and T_A_ show a strong temperature dependence and vanish completely at room temperature (not shown).

The ODMR measurements performed at *T* = 5 K clearly demonstrate that triplet excitons are formed on donor and acceptor molecules in the neat materials and in blends. However, to determine whether they are also formed in working solar cells, additional EDMR measurements were done on a solar cell under realistic operating conditions (300 K, 1 sun illumination intensity, open circuit conditions) and are shown in the same figure (black line). Although the magnetic resonance effect causes the *V*_OC_ to decrease, the shape of the spectrum (and its turning points) clearly shows the presence of PC_71_BM triplet excitons, but no sign of PTB7 triplet excitons^62^.

## Discussion

To understand the triplet formation loss pathway in PTB7:PC_71_BM blends, we first estimate the energetic positions of the relevant states. Exact measurements of the energies of the triplet exciton and CT states is difficult because triplet excitons are not optically active in most organic semiconductors, and the absorption and emission cross sections of the CT states are very weak. However, several well-tested empirical relationships have been determined to be very effective at estimating the state energies. The triplet exciton state has been found to consistently lie about 0.6–0.7 eV below the singlet state in semiconducting polymers^63^,^64^,^65^, and the singlet state can be estimated using the midpoint between the low energy peak of the absorption spectra and the high energy peak of the PL spectra^7^. Based on previous optical measurements^58^, we estimate the PTB7 singlet exciton at 1.65 eV and the triplet exciton energy at 1 eV. For PC_71_BM, the triplet exciton state is weakly phosphorescent and can be estimated from the PL spectrum^23^,^25^. With an estimated reorganization energy of 0.1–0.2 eV and a PL peak at 1.55 eV^23^, the PC_71_BM triplet exciton state is estimated at 1.6–1.7 eV. The intermolecular CT states also have both singlet and triplet types (^1^CT and ^3^CT respectively), but due to the large electron-hole separation distance, the singlet-triplet energy splitting is very small and the two states are commonly assumed to be degenerate^66^. The ^1^CT state energy can be estimated directly from the open-circuit voltage due to the well characterized relationship in which the ^1^CT state energy is about 0.5–0.6 eV higher than qV_OC_^67^,^68^,^69^. With an open-circuit voltage at around 0.75 eV^48^,^70^,^71^, the ^1^CT state energy is estimated to be 1.3 eV, which agrees with a corresponding PL band emerging for the blend films at low temperatures as shown in Fig. 1 (left).

**Figure 2:**
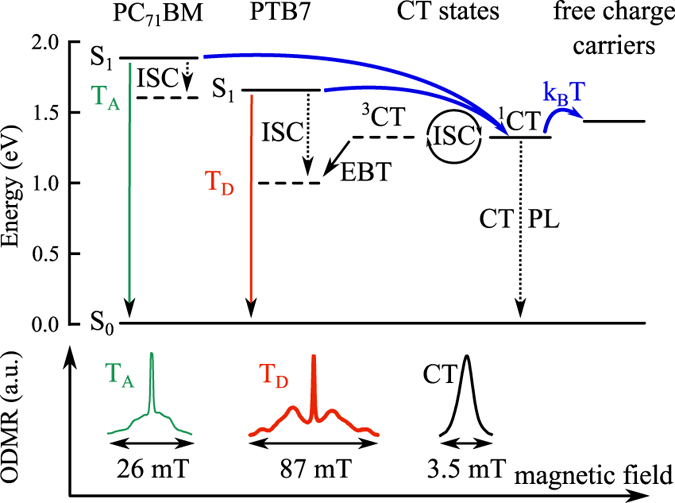
Top: Jablonski diagram depicting the relevant electronic states in the PTB7:PC_71_BM blend, ground states (S_0_), singlet exciton states (S_1_), PTB7 (donor) triplet exciton state (T_D_), PC_71_BM (acceptor) triplet exciton state (T_A_), singlet charge transfer state (^1^CT), triplet charge transfer state (^3^CT) and free charge carriers. Bottom: Typical ODMR spectra for T_A_, T_D_ und CT states and their spectral widths as distinctive features.

The resulting Jablonski state diagram based on these estimates is shown in Fig. 2, and this analysis suggests that PTB7 triplet exciton formation should be energetically favorable. In an optimized blend, exciton dissociation to the charge transfer states dominates over intersystem crossing^37^,^38^. As a result, triplet excitons on PTB7 are primarily expected to form via EBT, as shown in Fig. 2. In contrast, PC_71_BM triplet excitons are only expected to form via ISC from PC_71_BM singlet excitons because there is no other energetically favorable pathway.

Now we must consider the dominant processes occurring in the PTB7:PC_71_BM blends, and the factors that dictate them. With an excitation wavelength of 532 nm, singlet excitons (S_1_) are created on both PTB7 (donor) and PC_71_BM (acceptor) molecules. At 5 K, we assume that the excitons diffuse very little, and only the excitons created near the interface are able to access the ^1^CT state. As a result, in the blend without DIO, singlet excitons created in the interior of the large fullerene domains undergo a significant amount of intersystem crossing to the triplet exciton state (T_A_), as indicated by the ODMR measurements. This finding is also consistent with time resolved PL spectra of PTB7 blends with and w/o DIO that showed PC_71_BM singlet lifetimes of 180 ps and 518 ps for the blends with and w/o DIO, respectively^72^.

For the excitons that do dissociate, once in the ^1^CT state, there can be either a transition to the ^3^CT state or recombination to the ground state. A direct transition between ^1^CT and ^3^CT requires intersystem crossing, which can be slow^14^,^17^,^36^,^61^, but it has also been proposed that the CT states are weakly bound and that spin mixing could be mediated by charge separated states^11^,^17^. Finally, electron back transfer (EBT) can occur from the ^3^CT state to the donor triplet state (T_D_). In the blend without DIO, only a very small PTB7 triplet exciton signal is observed. This indicates that in general EBT is the dominant pathway to populate T_D_ and formation via ISC is less probable. Furthermore, the differences in the triplet signal between the two blends shows that EBT is indeed significantly affected by the fullerene domain size at low temperature, as previously hypothesized.

In addition to a change in domain size, an additional explanation to consider is a change in state energetics by adding DIO. DIO can also decrease the open-circuit voltage by about 0.05 V^51^,^71^,^73^, which could correspond to a modified ^1^CT state energy. As a result, if the triplet exciton state energy stays the same, the energetic driving force for EBT triplet formation could be decreased. However, the open-circuit voltage has also been correlated with the interfacial area^74^, and the DIO should also increase the interfacial area, which could also cause a decrease in the open-circuit voltage. In addition, the energy of the singlet state has also been shown to depend on the molecular order in the polymer, with higher order leading to a decreased singlet energy^75^. Fullerene energy levels also change due to crystallization^76^. However, previous morphology measurements have indicated that the addition of DIO does not cause significant crystallization or ordering of the mainly amorphous phases^51^. Based on this analysis, we find it unlikely that DIO causes significant enough changes to the energetics of the CT and triplet exciton states to cause the observed difference in triplet exciton yield.

While PTB7 triplet excitons are detected by PL at low temperatures, none can be found by ODMR or EDMR at 300 K. However, fullerene triplets and CT states become clearly visible at 300 K in the photovoltage (EDMR) measurement. This observation is consistent with the presence of fullerene clusters, as described by Hedley *et al*.^58^ In these clusters, some of the singlet excitons either fluoresce (as observed by the PL spectrum) or undergo intersystem crossing to T_A_, instead of dissociating into free charge carriers. The lack of PTB7 triplet excitons and the strong CT peak indicates that separation of the CT states kinetically out-competes EBT at 300 K, even when the fullerene domains are small as result of the DIO additive.

## Conclusion

Using spin-sensitive measurement techniques, we show that triplet exciton formation in PTB7:PC_71_BM blends is strongly influenced by morphology/sample treatment and temperature. ODMR measurements at 5 K indicate that the addition of DIO dramatically increases triplet exciton formation via EBT, presumably due to a reduced fullerene domain size. Careful energetic assessment shows that EBT should be an energetically favorable process. However, EDMR measurements on the blend with DIO in a working solar cell at 300 K show no signs of triplet exciton formation via EBT. As a result, these measurements show that EBT and triplet exciton generation in general are not major loss mechanisms in this particular solar cell material system. Therefore, tuning the domain size for optimal exciton harvesting and charge transport is more important than for reducing triplet exciton formation. In general, we can further assert that low energy offset blends are not inherently limited by triplet exciton loss pathways. Under the right conditions, even when triplet exciton formation is energetically favorable, EBT can be suppressed and high performance solar cells can be produced, as demonstrated by the PTB7:PC_71_BM blend with DIO. Further work understanding precisely why triplet exciton formation is suppressed in PTB7:PC_71_BM blends but not in some other blends will be particularly important for designing the next generation of high performing molecules for OPVs.

## Methods

The PTB7 was purchased from 1-Material, the PC_71_BM from Solenne, and the DIO from Sigma Aldrich, and all were used without additional purification. All materials were dissolved in chlorobenzene with a concentration of 20 mg/mL (PTB7) and 30 mg/mL (PC_71_BM), and 3 vol% DIO was added. Sample preparation was done in nitrogen atmosphere, and all thin films were manufactured by spin casting (60 s @ 800 rpm, 10 s @ 2500 rpm) on Herasil glass substrates, yielding about 100 nm thick films. These fabrication conditions have yielded films which achieve 7% power conversion efficiency in devices in our lab^70^,^71^.

For PL spectroscopy, the samples were mounted on a helium cold finger cryostat and kept under dynamic vacuum at *T* = 5 K or *T* = 300 K. The excitation source was a mechanically chopped cw laser at 532 nm with a power of 14 mW, and the PL was collected by large diameter concave mirrors and focused onto the entrance slit of a Cornerstone monochromator. The signal measured by a Si photodiode was recorded by a Signal Recovery 7265 DSP lock-in amplifier and then corrected for the spectral responsivity of the setup. The neat PTB7 and blend film samples were all 100 nm thick. However, the neat PC_71_BM sample had an undefined thickness due to crystallite formation.

For ODMR measurements, the substrates were sealed inside EPR quartz tubes (Wilmad) at 20 mbar helium atmosphere. Measurements were performed using a modified X-Band EPR spectrometer (Bruker 200D). The sample was placed in an optical resonant cavity (Bruker ER4104OR) equipped with a continuous flow helium cryostat (Oxford ESR 900). All measurements were recorded at *T* = 5 K with the sample surface being adjusted parallel to the external magnetic field and perpendicular to the 532 nm laser excitation beam with a power of about 40 mW. Microwaves were generated by a frequency synthesizer (Wiltron 69137A) and a microwave amplifier (Microsemi), with 1–2 W arriving at the cavity. PL from the films was collected with a Si photodiode, excluding the excitation wavelengths by a combination of 534 nm and 564 nm longpass filters. The preamplified (Femto DLPCA-200) variation of the photodiode current due to resonant microwave irradiation was recorded by a lock-in amplifier, referenced by TTL-modulating the microwave in the kHz range.

For EDMR measurements, the same setup was used with some modifications: The solar cell (glass, ITO, PEDOT:PSS, blend film with 3% DIO, Ca/Al) was illuminated with a white LED with intensity of 1 sun. The EPR-induced relative changes of the open circuit voltage (*V*_OC_) were recorded with lock-in at *T* = 300 K.

## Additional Information

**How to cite this article**: Kraus, H. *et al*. Analysis of Triplet Exciton Loss Pathways in PTB7:PC_71_BM Bulk Heterojunction Solar Cells. *Sci. Rep.* **6**, 29158; doi: 10.1038/srep29158 (2016).
